# QCM-D Investigations of Anisotropic Particle Deposition Kinetics: Evidences of the Hydrodynamic Slip Mechanisms

**DOI:** 10.1021/acs.analchem.2c01776

**Published:** 2022-07-01

**Authors:** Zbigniew Adamczyk, Agata Pomorska, Marta Sadowska, Małgorzata Nattich-Rak, Maria Morga, Teresa Basinska, Damian Mickiewicz, Mariusz Gadzinowski

**Affiliations:** † Jerzy Haber Institute of Catalysis and Surface Chemistry, Polish Academy of Sciences, Niezapominajek 8, Krakow 30 - 239, Poland; ‡ Centre of Molecular and Macromolecular Studies, Polish Academy of Sciences, Henryka Sienkiewicza 112, Lodz 90-363, Poland

## Abstract

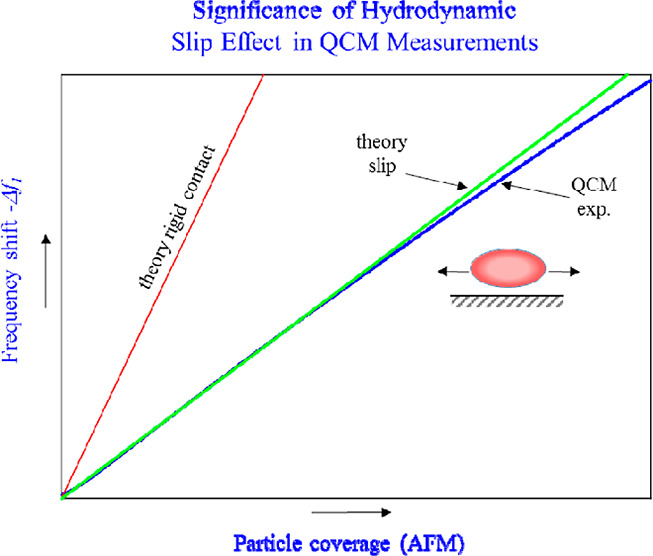

Deposition kinetics of positively charged polymer microparticles, characterized by prolate spheroid shape, at silica and gold sensors was investigated using the quartz microbalance (QCM) technique. Reference measurements were also performed for positively charged polymer particles of spherical shape and the same mass as the spheroids. Primarily, the frequency and bandwidth shifts for various overtones were measured as a function of time. It is shown that the ratio of these signals is close to unity for all overtones. These results were converted to the dependence of the frequency shift on the particle coverage, directly determined by atomic force microscopy and theoretically interpreted in terms of the hydrodynamic model. A quantitative agreement with experiments was attained considering particle slip relative to the ambient oscillating flow. In contrast, the theoretical results pertinent to the rigid contact model proved inadequate. The particle deposition kinetics derived from the QCM method was compared with theoretical modeling performed according to the random sequential adsorption approach. This allowed to assess the feasibility of the QCM technique to furnish proper deposition kinetics for anisotropic particles. It is argued that the hydrodynamic slip effect should be considered in the interpretation of QCM kinetic results acquired for bioparticles, especially viruses.

## Introduction

Investigations of particle deposition furnish essential information about their interactions with interfaces and about the adhesion strength, which is a crucial issue for colloid science, biophysics, medicine, soil chemistry, and so forth. This knowledge can be exploited to control and optimize various practical processes such as filtration, flotation, protective coating formation, catalysis, and microelectronics: production of nano- and microstructured materials such as narrow-band optical filters, photonic band gap materials, waveguides, and other electro-optical or magneto-optical devices.

It is also worth mentioning that the results acquired for model colloid systems can be exploited to properly interpret macromolecule adsorption phenomena, comprising protein molecules involved in thrombosis, artificial organ failure, plaque formation, and so forth.

One should consider that the shape of many colloid particles, for example, multiwalled carbon nanotubes,^1,2^ silica particles,^3−6^ polymer microparticles,^7−9^ polymer macroions (polyelectrolytes),^10−13^ and bioparticles such as DNA fragments,^14−16^ viruses,^17−19^ and bacteria,^20,21^ significantly deviates from the spherical shape and often resembles prolate spheroids or cylinders.

Because of its significance, nanoparticle and microparticle adsorption kinetics was extensively studied by a variety of experimental techniques such as optical microscopy,^22^ atomic force microscopy (AFM),^23−25^ scanning electron microscopy (SEM),^25−28^ ellipsometry,^29−31^ reflectometry,^32,33^ surface plasmon resonance,^34^ and electrokinetic methods.^35,36^ However, these techniques cannot provide valid information about the adhesive contact strength between the particles and the substrate surfaces. In this respect, the quartz crystal microbalance (QCM) method exhibits pronounced advantages, enabling precise, in situ deposition/desorption kinetic measurements for the nano- and microparticles under flow conditions.^37−51^ However, these investigations were almost exclusively focused on spherical particles.

In ref (43), the deposition of silica particles functionalized by silane from their suspensions in ethanol was investigated. It was observed that the frequency shift systematically increased whereas the dissipation shift decreased with the overtone number. These primary results were transformed to the dependence of the frequency shifts on the surface concentration of particles derived from SEM. It is shown that the slopes of these dependencies for all overtones were larger than the slope of the reference line pertinent to the dry coverage calculated using the Sauerbrey equation.

Using the QCM method, Olsson et al.^41^ investigated the deposition of 1 μm diameter silica particles at bare, biotinylated, and streptavidin-coated silica sensors. For the bare silica sensor, negative frequency shifts were only observed for overtone numbers below 9 and for ionic strength exceeding 0.05 M. For the streptavidin-coated silica, the frequency shift was strongly negative and linearly decreased with the overtone number. These results were interpreted in terms of the coupled resonance model, considering the damped oscillations of the particles attached to the sensor.

Tarnapolsky and Freger^49^ carried out thorough measurements of the deposition of polystyrene and silica microparticles at gold and silica sensors. The experimental data were expressed as the dependence of the frequency and bandwidth shift on the overtone number. The deposition of the 1 μm diameter polystyrene particles at a gold sensor produced negative frequency shifts, but the same particles at a silica sensor produced positive frequency shifts decreasing with the overtone number. This behavior was interpreted assuming that the low adhesion force for the silica particles/silica sensor (both bearing negative charge) enabled their oscillatory motion. On the other hand, for the same silica particle deposition at the positively charged sensor, the frequency shift was negative in accordance with the model where the deposited particles were assumed to oscillate in a stagnant fluid layer.

In ref (50), the deposition kinetics of positively charged amidine particles of size equal to 67, 140, 360, and 810 nm at Si/SiO_2_ sensors was studied by QCM. The primary frequency shifts versus the time dependencies were converted to the QCM coverage Γ_Q_ using the Sauerbrey equation and compared with the theoretical calculations derived from the random sequential (RSA) approach. It was confirmed that the QCM coverage was significantly larger than the dry particle coverage derived from ex situ AFM measurements denoted by Γ*.* In consequence, the Γ_Q_/Γ ratio attained 10 for the 67 nm diameter particles and the first overtone (in the short time limit). However, for the 810 nm diameter particles, the frequency shift rapidly decreased (in absolute terms) with the overtone number, yielding the QCM coverage much smaller than the inertia mass of the particle layer. This effect was attributed to the insufficient adhesion strength compared to the hydrodynamic force, which enabled a rocking motion of particles, albeit with no desorption.

Few QCM studies were performed for nonspherical particles. In refs (47) and (48), the deposition of liposomes at a titanium oxide sensor was investigated and theoretically interpreted in terms of lattice Boltzmann numerical modeling. It was predicted that the negative frequency shift should decrease with the particle coverage and the axis ratio of the liposomes modeled as oblate spheroids with the size equal to 78 nm. These theoretical calculations enabled to elaborate a unique procedure for the determination of the liposome aspect ratio under in situ conditions.

Scarce experiments were reported for colloid particles of elongated shape. In ref (52), the deposition of bullet-like silica particles with the dimensions 1100 × 250 nm (aspect ratio approximately equal to 4.5) at silica sensors was studied by QCM. Both the particles and the sensors exhibited negative ζ potentials for a broad pH range that resulted in a rather minor deposition efficiency characterized by the frequency shift amounting to −30 Hz. However, no attempt at the interpretation of these results was undertaken in ref (52).

Because of the deficit of experimental results, the main goal of this work was to determine the mechanism of spheroidal particle deposition at the solid/electrolyte interface, with the main focus on assessing the applicability of the QCM technique to yield quantitative kinetic results. In order to increase the reliability of experiments, monodisperse polymer particles of positive surface charge were used, which enabled their irreversible adsorption at negatively charged silica and gold sensors. The primary QCM data were compared with AFM measurements that provided the absolute particle coverage as a function of deposition time. This enabled a quantitative interpretation of the QCM results in terms of the theoretical model where the hydrodynamic slip effect promoting sliding particle motion was considered.

One can expect that the acquired results can be exploited as useful reference systems for a quantitative interpretation of protein and virus adsorption/desorption phenomena^51,53,54^ and for the estimation of the validity of the rigid and soft adhesion models.

## Experimental Section

### Materials

All chemical reagents comprising sodium chloride, sodium hydroxide, and hydrochloric acid were commercial products of Sigma-Aldrich and were used without additional purification. Ultrapure water was obtained using the Milli-Q Elix & Simplicity 185 purification system from Millipore.

### Synthesis of P(S/PGL) Spheroidal Microparticles

The synthesis of poly(styrene/α-*tert*-butoxy-ω-vinylbenzyl-polyglycidol) (PS/PGL) spheroidal microparticles was described in ref (55). It was a tedious process consisting of four main steps: (i) synthesis of α-*tert*-butoxy-ω-vinylbenzyl-polyglycidol (PGL) macromonomer, (ii) synthesis of P(S/PGL) microspheres using styrene and PGL macromonomer, (iii) preparation of spheroidal particles P(S/PGL) from the spherical ones, applying the stretching of poly(vinyl alcohol) (PVA) films containing embedded P(S/PGL) microspheres. After completing these three steps, one obtained uncharged spheroidal particles capped with a poly(vinyl alcohol) (PVA) surface layer. Finally, in the fourth step, positively charged particles were produced by a surface modification process consisting in the selective oxidation of surface hydroxyl to carboxyl groups and the consecutive adsorption of polyethyleneimine (PEI). The particle chemical composition was characterized by X-ray photoelectron spectroscopy (XPS) experiments, performed using the PHl 5000 VersaProbe–Scanning ESCA Microprobe (ULVAC-PHI, Japan/USA) instrument at a base pressure below 5 × 10^–9^ mbar. The particle morphology and size distribution were characterized by SEM using a JEOL 5500LV apparatus (Akishima, Japan).

Except for the spheroidal PS/PGL, positively charged amidine polystyrene microparticles supplied by Invitrogen were used in the measurements of the deposition kinetics carried out by QCM.

The concentration of particles in the stock suspension was determined by densitometry and the dry mass method. Before each deposition experiment, the stock suspension was diluted to the desired concentration, typically equal to 200–300 mg L^–1^, by pure NaCl solutions, with the pH adjusted to 5.6.

The diffusion coefficient of microparticles was determined by dynamic light scattering (DLS) using the Zetasizer Nano ZS instrument from Malvern. The hydrodynamic diameter was calculated using the Stokes–Einstein relationship. The electrophoretic mobility of particles was measured by the laser Doppler velocimetry (LDV) technique using the same apparatus. The ζ-potential was calculated using the Henry formula.^28^

Quartz/silica plates were used in the streaming potential measurements. They were cut off from silicon wafers supplied by ON Semiconductor. Before each measurement, the plates were oxidized in a controlled way using a mixture of 96% sulfuric acid (H_2_SO_4_) and hydrogen peroxide (30%) in 1:1 volume ratio for 30 min. Afterward, they were rinsed by deionized water and boiled at 80 °C for 30 min, rinsed with ultrapure water and dried out in a stream of nitrogen gas.

Gold plates used in the streaming potential measurements were prepared using Si/SiO_2_ wafers as the supporting substrate, which were coated with a gold layer (approximately 100 nm thick) by thermal evaporation. The gold-coated wafers were cleaned by applying the same procedure as that was used for quartz/silica dioxide substrates.

Quartz/silica dioxide (SiO_2_) and gold sensors used in the experiments were supplied by Q-Sense, Gothenburg, Sweden. Before measurements, the sensors were cleaned in a 30 minute old mixture of 96% sulfuric acid (H_2_SO_4_), hydrogen peroxide (30%), and ultrapure water in a volume ratio of 1:1:1 for 3 min. Afterward, the sensors were rinsed with ultrapure water and boiled at 80 °C for 30 min. Finally, they were rinsed again with ultrapure water and dried out in a stream of nitrogen gas. The sentence should read: The roughness of the sensors was examined by AFM in the semi-contact mode under ambient conditions. It was confirmed that the sensors exhibited the root-mean-square (rms) roughness below 1 nm.

### Methods

The QCM measurements were carried out according to the standard procedure described in ref (50) using the Q-Sense QCM instrument (Biolin Scientific, Stockholm, Sweden). Initially, a stable baseline for the pure electrolyte (NaCl) of controlled ionic strength and pH was obtained. Then, particle suspension of controlled concentration was flushed through the cell at a fixed flow rate. After a prescribed time, the desorption run was initiated where a pure electrolyte solution of the same pH and ionic strength was flushed through the cell.

The deposition kinetics of particles was independently determined using the AFM method, as previously described in ref.^50^ Accordingly, the QCM adsorption runs were stopped after discrete time intervals, and the sensors were removed from the suspension and imaged under ambient conditions by AFM using the NT-MDT Solver BIO device with the SMENA SFC050L scanning head. The number of particles per unit area (typically 1 μm^2^), denoted hereafter by *N,* was determined by a direct counting of over a few equal-sized areas randomly chosen over the sensor, with the total number of particles exceeding 2000.

The ζ potential of the plates was determined via the streaming potential measurements performed according to the procedure described in ref (36), applying the Smoluchowski formula where the correction for surface conductivity was considered.

All experiments have been performed at the temperature of 298 K.

The deposition kinetic runs derived from AFM were theoretically interpreted in terms of a hybrid approach where the bulk particle transport was described by the convective diffusion equation, with the nonlinear boundary condition derived from the RSA model (a detailed description of this approach is given in the Supporting Information).

## Results and Discussion

### Bulk Particle and Substrate Characteristics

**Figure 1:**
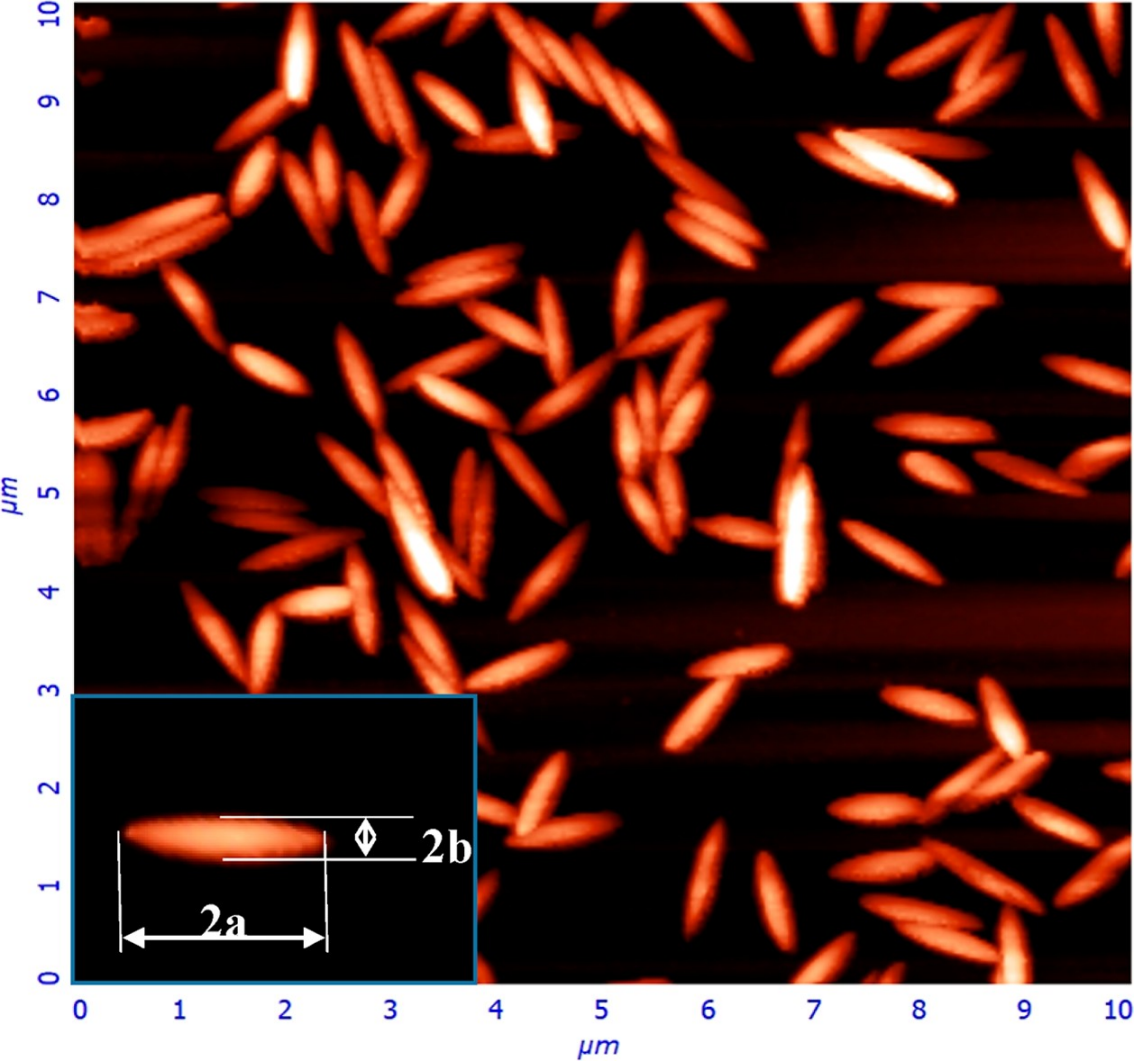
AFM image of the (PS/PGL) spheroidal particles deposited at the silica sensor; the surface concentration of the layer is equal to 1.23 μm^–2^. The inset shows a single spheroid with the definitions of its dimensions.

The (PS/PGL) particle dimensions were determined from AFM images, and a typical micrograph is shown in Figure 1.

Briefly, histograms of particle length, denoted hereafter by 2*a*, and width, denoted by 2*b*, were separately obtained by measuring the corresponding dimensions of ca. 200 particles using an image analyzing software. It was determined in this way that the average length of particles was equal to 1020 ± 20 nm, and the width was equal to 220 ± 10 nm (see Table 1). This indicates that the particles were fairly monodisperse, and their shape could well be approximated by a prolate spheroid shape. Accordingly, their axis ratio λ = *a/b* was equal to 4.64, and the cross-sectional area in the side-on orientation was equal to 1.76 × 10^5^ nm^2^ (0.176 μm^2^) (see Table 1). Using these dimensions, one can calculate the hydrodynamic diameter of the particle *d*_H_ from the analytical formula^56^
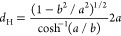
1

**Table 1:** Physicochemical Characteristics of the P(S/PGL) Particles Investigated in This Work

property, symbol	value	remarks
density [g cm–^3^], ρ_*p*_	1.06 ± 0.02 g cm–^3^	dilution method
size, shape	2*a* × 2*b* × 2*b*	AFM
	1020 ± 20 × 220 ± 10 × 220 ± 10 nm	
	1.02 × 0.220 × 0.220 μm	
geometrical cross-sectional area, Sg	0.176 μm^2^	side-on orientation of the particle
	0.0380 μm^2^	end-on orientation of the particle calculated from AFM dimensions
particle mass, *m*_1_	2.74 × 10^–14^ g	from particle size and density
axis ratio, λ = a/b	4.64	from the particle geometry
diffusion coefficient, D	1.12 × 10^–8^ cm^2^ s^–1^	DLS measurements, T = 298 K, pH range 4–9, NaCl concentration range 10^–3^–10^–2^ M
hydrodynamic diameter, *d*_H_	0.440 ± 0.020 μm	derived from DLS measurements
	0.450 μm	calculated from the particle geometry eq 1
equivalent sphere diameter, *d*s	370 nm	calculated as 2(*ab*^2^)^1/3^
	0.370 μm	
electrophoretic mobility, μ_e_	3.2 ± 0.1 μm cm (V s)^−1^	from LDV measurements, pH 5–6, NaCl concentration 10^–3^–10^–2^ M
ζ potential, ζ	40 ± 2 mV	calculated from the Henry equation, pH 5–6, NaCl concentration 10^–3^–10^–2^ M

It was equal to 450 nm (0.450 μm), which is larger than the equivalent sphere diameter of 370 nm, calculated using the formula 2(*ab*^2^)^1/3^.

Independently, the hydrodynamic diameter of the spheroidal particles was determined from the DLS measurements, directly yielding the diffusion coefficient for the microparticles. It was equal to 1.12 ± 0.02 × 10^–8^ cm^2^ s^–1^ (for the pH range 4–9 and the NaCl concentration 10^–3^ to 10 ^–2^ M). Considering this value, the hydrodynamic diameter of 440 ± 20 nm was obtained using the Stokes–Einstein formula.

The ζ potential of the particles derived from the LDV measurements was equal to 40 ± 2 mV for the NaCl concentration 10^–3^ to 10^–2^ M and pH 5–6.

In an analogous way, the amidine particles were characterized by DLS measurements, thus yielding their diffusion coefficient equal to 1.3 ± 0.02 × 10^–8^ cm^2^ s^–1^ (for the pH range 5–6 and the NaCl concentration 10^–3^ to 10^–2^ M. This corresponds to the hydrodynamic diameter equal to 370 ± 0.010 nm, which coincides with the equivalent sphere diameter pertinent to spheroids. Given the amidine particle density equal to 1.05 ± 0.02 g cm^–3^, one can deduce that the masses of the spheroidal and the spherical particles were practically the same. The ζ potential of the amidine particles was equal to 58 ± 4 mV for the NaCl concentration of 10^–2^ M and pH 5–6.

It should be mentioned that the particle suspensions were stable over the time period significantly exceeding the time of the typical QCM experiments. This was confirmed in separate experiments via the DLS measurements where the particle size distribution was monitored as a function of the storage time.

The ζ potential values of the oxidized Si/SiO_2_ substrate determined by the streaming potential measurements were equal to −45 and −60 mV for the ionic strength values of 10^–2^ and 10^–3^ M, respectively, at pH 5–6.

The ζ potential values calculated for gold substrates were equal to −47 and −58 mV for the ionic strength values of 10^–2^ and 10^–3^ M, respectively, at pH equal to 5–6.

### Kinetics of Particle Deposition

One should mention that the use of polymer nanoparticles in the QCM studies is advantageous because of their positive ζ potential opposite to the sensor ζ potential that promotes an irreversible deposition regime.^50^ Also, a precise determination of the surface concentration for this particle size range is feasible using AFM, which enables a quantitative analysis of the obtained results.

**Figure 2:**
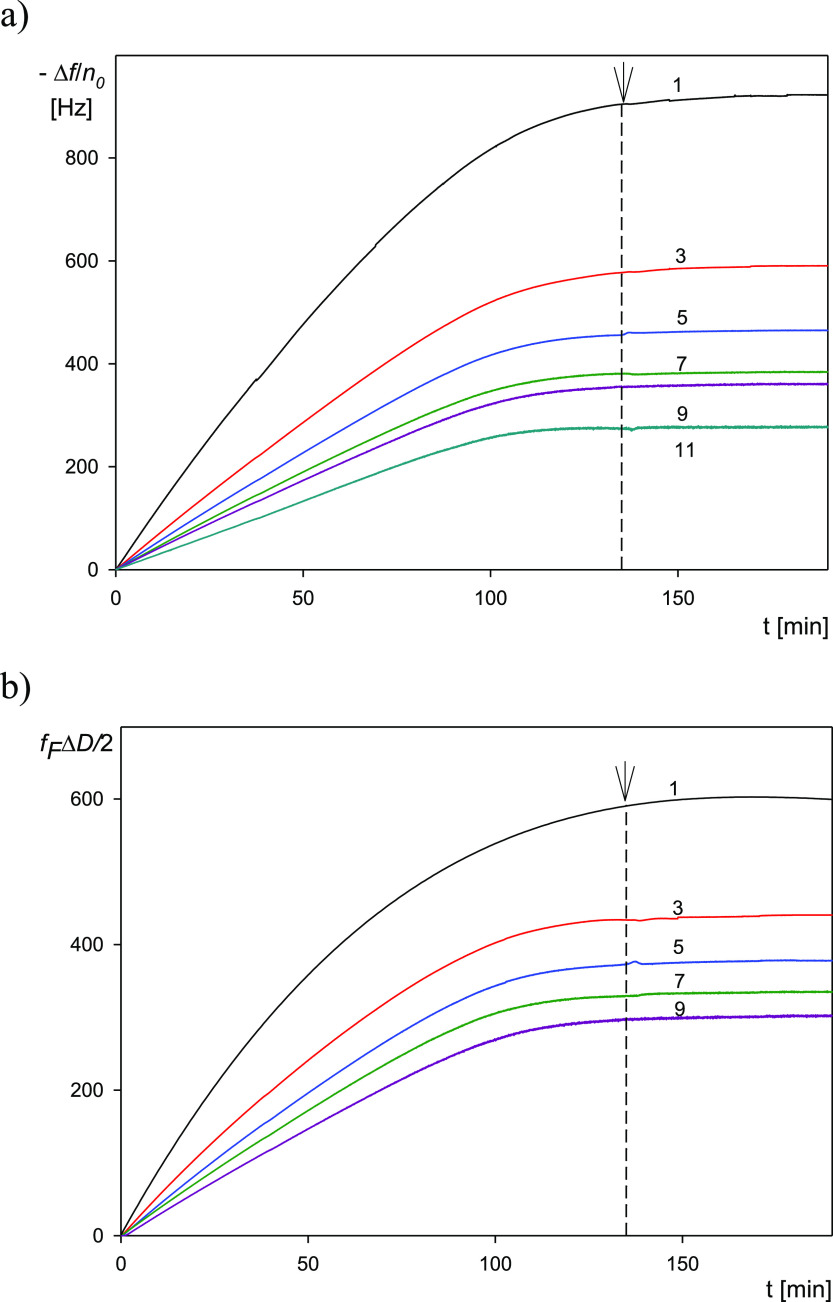
(a) Normalized frequency shift −Δ*f/n*_0_ vs deposition time for various overtones *n*_0_ = 1–11 derived from QCM measurements; (b) normalized bandwidth shift *f*_F_Δ*D*/2 for various overtones *n*_0_ = 1–9. Experimental conditions: (PS/PGL) spheroidal particles, silica sensor; ionic strength, 0.01 M; pH, 5.6; volumetric flow rate, 10^–3^ cm^3^ s^–1^; and bulk particle concentration, 200 mg L^–1^. At the time of 140 min, the desorption run was initiated (denoted by the arrow), where the pure electrolyte was flushed through the cell.

A primary QCM kinetic run acquired for the (PS/PGL) spheroidal particles is shown in Figure 2. In part a of this figure, the normalized frequency shift −Δ*f*/*n*_0_ is plotted as a function of time for various overtones *n*_0_ = 1 to 11. Analogously, in part b, the dependence of the bandwidth shift *f*_F_Δ*D*/2^49^ on time for various overtones *n*_0_ = 1 to 9 is shown (where *f*_F_ is the fundamental frequency of the quartz resonator equal to 5 × 10^6^ Hz, and Δ*D* is the dissipation shift). Such a form of the presentation of experimental results is convenient for their interpretation in terms of the hydrodynamic model formulated in the Supporting Information

One can observe that −Δ*f*/*n*_0_ monotonically increases with time, attaining stationary values, which markedly differ among the overtones. Thus, for the adsorption time of 140 min, the normalized frequency shift values were equal to 900 and 280 Hz for the first and ninth overtones, respectively. Interestingly, these values do not change after switching to the desorption run where the pure electrolyte was flushed through the cell. This suggests an irreversible deposition of particles with minimum desorption.

An analogous behavior was observed for the bandwidth shift *f*_F_Δ*D*/2, which also monotonically increased with time, attaining stationary values equal to 600 and 270 Hz for the first and ninth overtones, respectively.

One should mention that such behavior where both −Δ*f*/*n*_0_ and *f*_F_Δ*D*/2 monotonically decrease with the overtone number was reported in ref (49) for negatively charged silica particle deposition at the macroion-modified silica sensor, whose charge was positive. The −Δ*f*/*n*_0_ values for the 1st and 11th overtones were equal to 280 and 100 Hz, respectively, whereas the bandwidth shift *f*_F_Δ*D*/2 values were equal to 400 and 55 Hz for these two overtones.

Our experimental results were interpreted in terms of the hydrodynamic model recently developed in refs (57) and (58) for spherical particles, where it is confirmed that hydrodynamic forces inducing particle slip over the sensor play an essential role. Accordingly, a thorough analysis of the forces acting on the particles deposited under the side-on orientation at the QCM sensor was carried out in the Supporting Information. A few measurement regimes were considered, and analytical expressions for the frequency and dissipation shifts were derived. In the case where deposited particles form a rigid contact with the sensor, the normalized frequency and bandwidth shifts were described by the formula
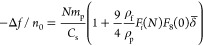
2
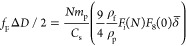
where *C*_s_ = *Z*_q_/2*f*_F_^2^  is the Sauerbrey constant equal to 0.177 (mg m^–2^ Hz^–1^) for *f*_F_ = 5 × 10^6^ Hz, *Z*_q_ is the acoustic impedance of the quartz sensor equal to 8.8 × 10^6^ kg m^–2^ s^–1^, *N* is the surface concentration of the deposited particles (number of particles per unit area of the sensor), *m*_p_ is the particle mass, ρ_f_ and ρ_p_ are the fluid and particle densities, respectively, δ̅ = δ/*b* is the normalized penetration depth (δ is the hydrodynamic layer thickness^49^), *b* is the particle characteristic dimension, *F*_*i*_(*N*) is the correction function accounting for ambient flow damping due to the deposited particles, which approaches unity in the low coverage range, and *F*_8_(0) is the hydrodynamic wall correction function calculated in refs (59) and (60).

The first term in the bracket in eq 2 corresponds to the normalized inertia, and the second term corresponds to the normalized hydrodynamic force.

It is to mention, however, that eq 2 remains accurate for δ̅>1. For the opposite case, where δ̅≤1, analogous expressions were derived in the Supporting Information.

In the case of neutrally buoyant particles which can freely translate and rotate, the inertia force cannot be transferred to the sensor; therefore, one obtains the following formula describing the frequency shift (Supporting Information)
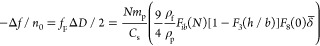
3where *h* is the equilibrium surface-to-surface distance between the particle and the sensor (gap width), corresponding to the primary minimum distance in the case of irreversible deposition;^54^*F*_*ib*_(*N*) is the correction function accounting for the particle coverage; and *F*_3_(*h*/*b*) is the hydrodynamic function describing the slip velocity of the particle relative to the oscillating fluid.^59^

Considering that δ = (2*v*/ω)^1/2^ = (*v*/π*n*_0_*f*_F_)^1/2^,^49^ one can infer from eq 3 that −Δ*f*/*n*_0_ = *f*_F_Δ*D*/2∼*n*_0_^–1/2^, which means that the normalized frequency and bandwidth shifts are equal (in absolute terms) and decrease as the square root of the overtone number.

However, one should mention that eq 3 is strictly valid for (*b* + *h*)/δ > 1. In the opposite case, where (*b* + *h*)/δ ≤ 1, analogous expressions were derived in the Supporting Information

It is also useful to define the following parameter expressing the ratio of the normalized frequency to the bandwidth shift
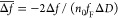
4

Thus, for the rigid contact in the limit of low coverage, one has
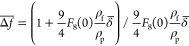
5

Therefore, for neutrally buoyant particles and (*b* + *h*)/δ > 1
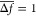
6

This formula indicates that the normalized frequency and the bandwidth signals derived from QCM measurements convey in the case of a neutrally buoyant particle equivalent information that was postulated in ref (62). This property directly stems from the linearity of the quasi-stationary Navier–Stokes equation governing the oscillatory flows around deposited particles in the limit of (*b* + *h*)/δ > 1 (Supporting Information).

It is expected that eq 6 is valid for arbitrary coverage, the overtone number, and the distance of the particle from the sensor.

**Figure 3:**
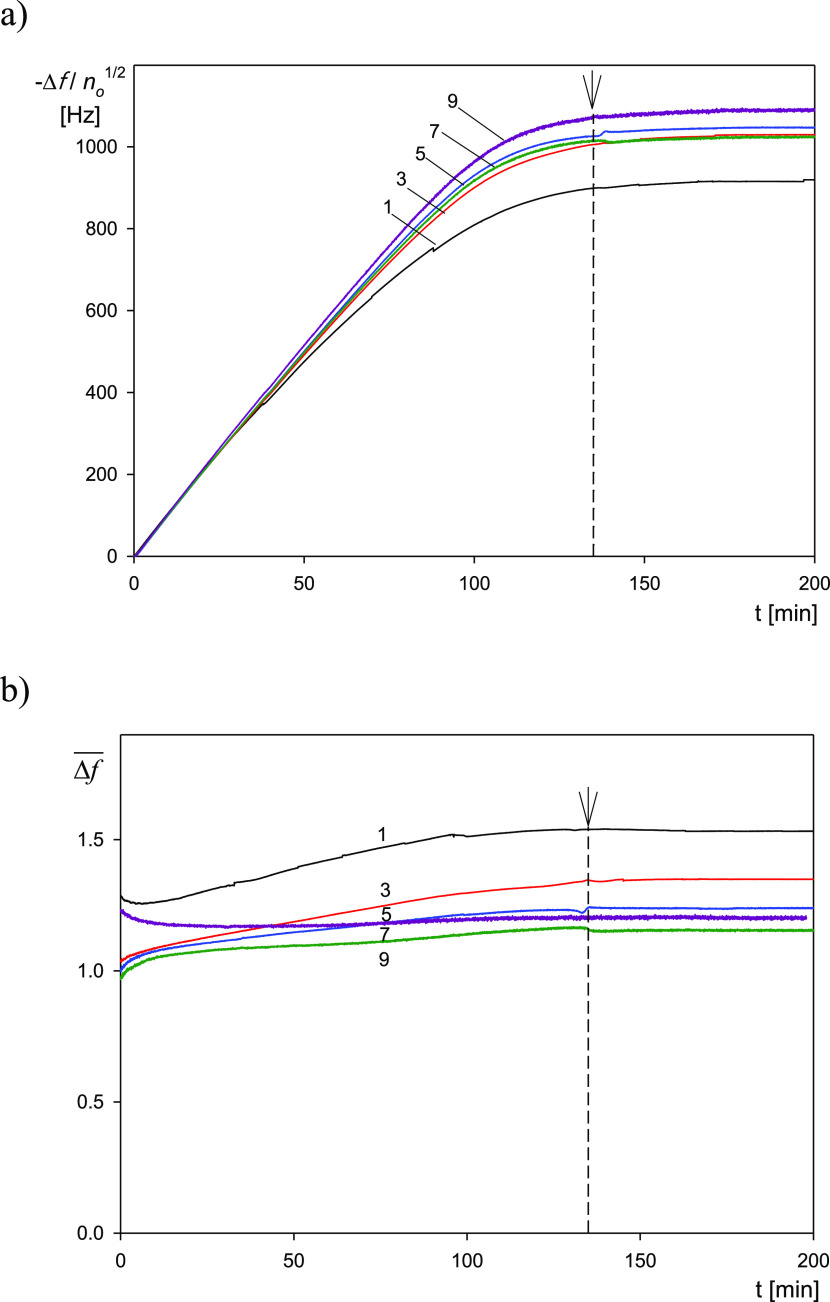
(a) Normalized frequency shift −Δ*f*/*n*_0_^1/2^ vs deposition time for various overtones *n*_0_ = 1–11 derived from QCM measurements. (b) Ratio of the normalized frequency to the bandwidth shifts Δ*f̅* = −2Δ*f*/(*n*_0_Δ*Df*_F_) for various overtones *n*_0_ = 1–9. Experimental conditions: (PS/PGL) spheroidal particles, silica sensor; ionic strength, 0.01 M; pH, 5.6; volumetric flow rate, 10 ^–3^ cm^3^ s^–1^; and bulk particle concentration, 200 mg L^–1^. At the time of 140 min, the desorption run (denoted by the arrow) was initiated, where the pure electrolyte was flushed through the cell.

Considering these theoretical predictions, the primary QCM results are presented in Figure 3 as the transformed frequency shift −Δ*f*/*n*_0_^1/2^ versus the deposition time for various overtones *n*_*o*_ = 1–11 (Figure 3a) and as the ratio of the normalized frequency to the bandwidth shift Δ*f̅* (Figure 3b).

One can observe in Figure 3a that for the deposition time below 100 min −Δ*f*/*n*_0_^1/2^ linearly increases for all overtones, with the common slope equal to 10.8 Hz s^–1^.

Hence, the results shown in Figure 3a confirm the trend predicted from eq 3 pertinent to the freely buoyant particle layer, where −Δ*f*/*n*_0_^1/2^ should be constant for all overtones (for the same deposition time). Moreover, according to eq 6, for freely buoyant particles, the ratio of the normalized frequency to the bandwidth shifts Δ*f̅* should remain constant and close to unity for all overtones, which is indeed observed in Figure 3b.

However, a quantitative check of the theoretical prediction is feasible if the particle mass coverage Γ = *Νm*_p_ (referred usually to as the dry coverage) is known as a function of the deposition time. As shown in the Supporting Information, at shorter deposition times, where the surface blocking effects are negligible, the coverage is a linear function of time, explicitly given by^61^
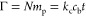
7where *k*_c_ is the mass transfer rate constant and *c*_b_ is the mass concentration of particles in the bulk.

**Figure 4:**
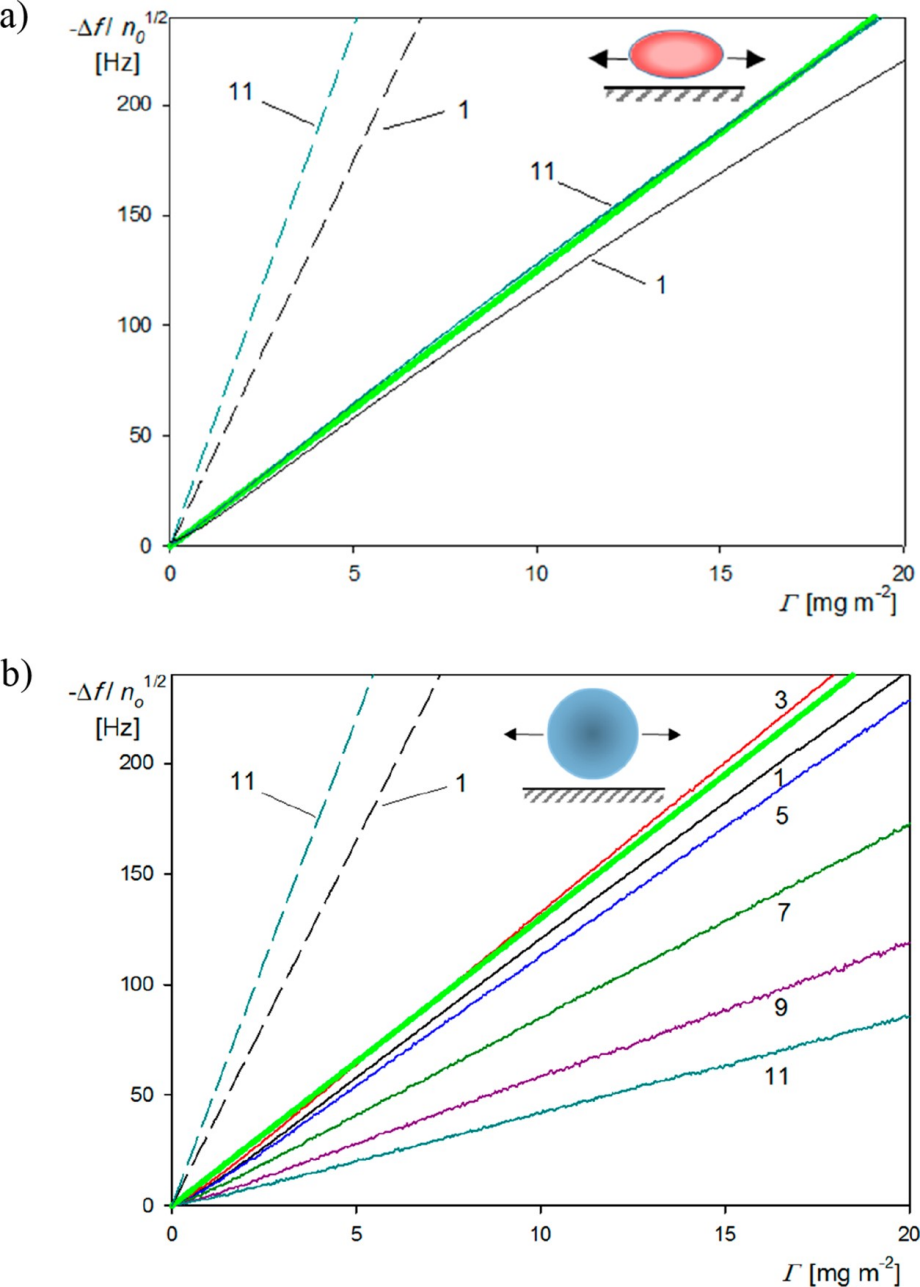
Normalized frequency shift −Δ*f*/*n*_0_^1/2^ vs the particle mass coverage for various overtones *n*_0_ = 1 and 11 derived from QCM measurements. (a) Spheroidal particles, silica and Au sensors; ionic strength, 0.01 M; volumetric flow rate, 10^–3^ cm^3^ s^–1^; and bulk particle concentration, 200 mg L^–1^. The solid green line shows the theoretical results calculated from eq 8 for the neutrally buoyant particles, and the dashed lines show the theoretical results predicted from eq 9 for rigidly fixed particles (for overtones 1 and 11). (b) Spherical particles, silica sensor; ionic strength, 0.01 M; volumetric flow rate, 3 × 10^–3^ cm^3^ s^–1^; and bulk particle concentration, 300 mg L^–1^. The solid green line shows the theoretical results calculated from eq 8 for the neutrally buoyant particles, and the dashed lines show the theoretical results predicted from eq 9 for rigidly fixed particles (for overtones 1 and 11).

Therefore, the mass transfer rate constant for each experiment carried out for different suspension flow rates, for SiO_2_ and Au sensors, was determined by ex situ AFM imaging of the adsorbed particle layer over various areas of the sensor. Experimental results obtained for the spheroidal particles and ionic strength of 0.01 M are shown in Figure 4a as the dependence of −Δ*f*/*n*_0_^1/2^ for the overtones 1 and 11 on the particle coverage calculated from eq 7. As can be seen, the experimental data in all cases are well approximated by linear dependencies, with a minimum difference among these two limiting overtones (the results for the overtones 3–9 were in between these limiting values and are not shown in Figure 4a for the sake of clarity).

The experimental results are adequately reflected by the theoretical model pertinent to neutrally buoyant particles executing a sliding motion (solid green line in Figure 4a), where the normalized frequency shift was calculated from the dependence
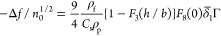
8The following parameters were used in these calculations: ρ_f_ = 1.0 kg m^–3^, ρ_p_ = 1.06 kg m^–3^, *F*_3_(*h*/*b*) = 0.57 (calculated for *h*/*b* = 0.02 in the Supporting Information), *F*_8_(0) = 1.14 (pertinent to the string of touching beads), and δ̅_1_ = 2.16. It is worth mentioning that, except for the above data, in these calculations, no other adjustable parameters were used. In contrast, the results predicted for the rigid contact, depicted by the dashed lines in Figure 4a, where the normalized frequency shift is given by
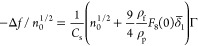
9many times overestimate the experimental data. Analogous experimental results obtained for the spherical particles (A360) are presented in Figure 4b. As previously observed for spheroids, the experimental data are well approximated by linear dependencies for all overtones (1–11), with the slopes similar for the first to fifth overtones, whereas for larger overtones, the dependencies of −Δ*f*/*n*_0_^1/2^ on the coverage were characterized by much smaller slopes. As before, the experimental results for the overtones 1–5 agree with those derived from the theoretical model pertinent to neutrally buoyant particles, using eq 8 (green line in Figure 4b), where the following parameters were used: ρ_f_ = 1.0 kg m^–3^ ρ_p_ = 1.06 kg m^–3^, *F*_3_(*h*/*b*) = 0.52, *F*_8_(0) = 1.70, and δ̅_1_ = 1.32. Again, as for the spheroids, the results predicted for a rigid particle contact and calculated from eq 9 significantly overestimate the experimental data. The significant deviation of experimental data from the theoretical results observed in Figure 4b for larger overtones (7–11) can be most likely explained as suggested in ref (49) in the appearance of elastic, van der Waals forces, which damp the oscillatory particle motion.

As one can infer from eq 8, the dry coverage of spheroidal particles can be calculated from the QCM signal using the linear dependence valid for all overtones
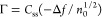
10where
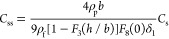
11is the modified Sauerbrey constant pertinent to the neutrally buoyant particle model.

It should be mentioned that the *F*_3_(*h*/*b*) function in eq 11 can well be approximated by the analytical interpolation given in the Supporting Information.

However, one should mention that eq 10 is expected to yield precise results if δ_1_/*b* > 1.

**Figure 5:**
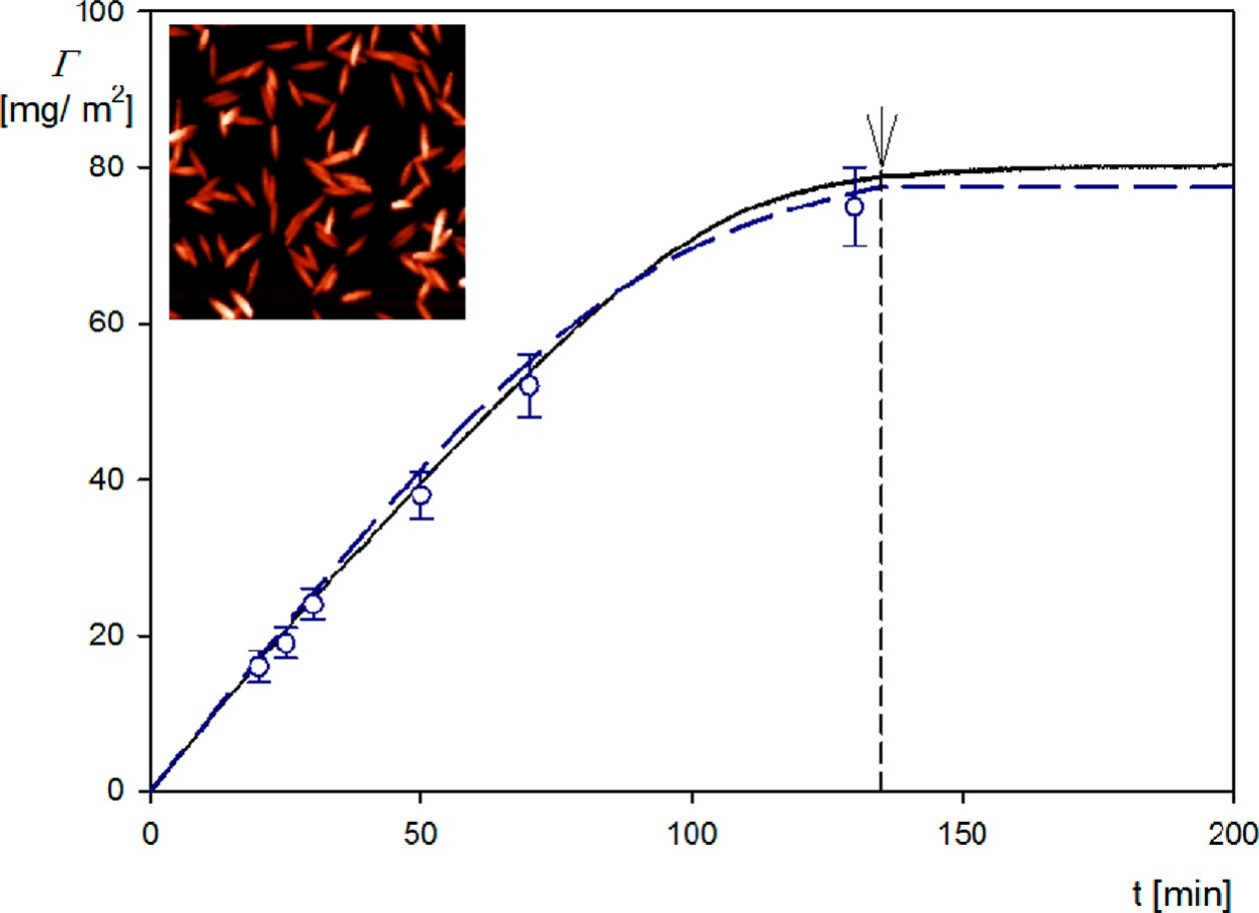
Kinetics of particle deposition expressed as the dependence of the mass coverage on the deposition time; the solid lines represent the QCM results calculated from eq 10, considering the slip effect [average value from overtones (1–11)]; the points represent the experimental dry coverage derived from AFM, and the dashed lines denote the theoretical results derived from the RSA model. Spheroidal particles, silica sensor; ionic strength, 0.01 M; volumetric flow rate, 10^–3^ cm^3^ s^–1^; and bulk particle concentration, 200 mg L^–1^. The inset shows the AFM micrograph of the particle layer.

In order to assess the validity of eq 10, the primary QCM kinetic runs, that is, the dependence of the frequency shift on the deposition time (see Figure 2), were transformed to the dependence of the dry coverage on time and shown in Figure 5.

As can be seen, the coverage calculated in this way as an average from the first to eleventh overtones (solid line in Figure 5) agrees with the experimental data derived from AFM, which directly yield the particle coverage *albeit* for discrete time intervals (these results are shown as points in Figure 5). The QCM results are also compared with the theoretical calculations derived from the hybrid RSA approach (Supporting Information), where the coupling of the bulk particle transfer with the surface transfer through the particle layer governed by blocking effects is considered in an exact way. The kinetic equation derived within the scope of this approach was numerically integrated, which explicitly yielded the particle dry mass versus adsorption time dependencies. This approach was previously used for a quantitative interpretation of nanoparticle deposition kinetics at various substrates.^54^ One can observe in Figure 5 that the RSA results agree with the QCM and AFM experimental data for the entire range of the deposition time. Therefore, these results confirmed that it was possible to quantitatively interpret the results of QCM measurements for anisotropic particles in terms of the theoretical model considering the hydrodynamic slip effect.

## Conclusions

Deposition kinetics of positively charged spheroidal microparticles at oppositely charged silica and gold sensors was quantitatively determined, applying the quartz microbalance (QCM) technique combined with the AFM measurements.

It was observed that the normalized frequency and bandwidth shifts for various overtones were equivalent, rendering their ratio close to unity for the entire deposition time. The normalized frequency shift was transformed to the dependence on the particle mass coverage, which was determined by AFM. The results expressed in this way were quantitative, interpreted in terms of the hydrodynamic model pertinent to neutrally buoyant particles where a significant slip effect was predicted. In the case of such a lubrication-like contact, the QCM signals become many times smaller than those expected for a rigid contact, even for the gap width amounting to 1% of the particle size.

Considering the hydrodynamic slip effect, a modified Sauerbrey-like equation was derived, enabling to calculate the absolute particle coverage from the frequency shift normalized by the square root of the overtone number. It is demonstrated that the QCM coverage predicted from this equation agrees with the AFM data and with the theoretical results derived from the RSA model.

One can expect that the hydrodynamic slip effect can also play a significant role for nanoparticle-sized solutes comprising virus capsids and proteins whose molecules are characterized by irregular shape. Such molecule topography can decrease the adhesion strength, especially for sensors characterized by pronounced roughness, which enables the solute sliding motion around the equilibrium position. Because in such cases the ratio of the gap width to the characteristic molecule dimension can exceed 0.1, a manyfold decrease in the QCM signal compared to the rigid contact is predicted.

## Supporting Information Available

Modeling QCM response and modeling adsorption kinetics of particles (PDF)
