# Supplementary material for: QCM-D Investigations of Anisotropic Particle Deposition Kinetics: Evidences of the Hydrodynamic Slip Mechanisms
Source: Anal Chem. 2022 Jul 1;94(28):10234–44. doi: 10.1021/acs.analchem.2c01776 (PMC9310025; doi:10.1021/acs.analchem.2c01776)
Supplement: Supplementary file 1 — ac2c01776_si_001.pdf [file ac2c01776_si_001.pdf]

SUPPORTING INFORMATION

QCM-D Investigations of Anisotropic Particle Deposition Kinetics: Evidences of the Hydrodynamic Slip Mechanisms

Zbigniew Adamczyk[1]*, Agata Pomorska[1], Marta Sadowska[1],
Małgorzata Nattich-Rak[1], Maria Morga[1]
Teresa Basinska[2], Damian Mickiewicz[2], Mariusz Gadzinowski[2],

1 Jerzy Haber Institute of Catalysis and Surface Chemistry, Polish Academy of Sciences, Niezapominajek 8, 30 - 239 Krakow, Poland;
2 Centre of Molecular and Macromolecular Studies, Polish Academy of Sciences, H. Sienkiewicza 112, 90-363 Lodz, Poland;

*Corresponding author, e-mail: zbigniew.adamczyk@ikifp.edu.pl

Table of contents:

1 QCM Response to a Heterogeneous (Particle-like) Load.

1.1 Basic Considerations

1.2 Force on rigidly fixed particles

1.3 Force on neutrally buoyant particles

2 Modeling Adsorption Kinetics of Particles in the QCM cell.

1 QCM Response to a Heterogeneous (Particle-like) Load.

1.1 Basic Considerations

The QCM sensor load for nano- and microparticle size range, typically equal to 20-2000 mg m^{-2} [1], is considerably lower than the quartz crystal surface density equal to 5×10^{6} mg m^{-2} (assuming the crystal thickness of 0.2 cm). In consequence, particle deposition occurs under the small-load (Sauerbrey) regime[2-4] where the complex QCM response is given by

$$\Delta f^{*} = \Delta f + \frac{f_F n_0}{2} \Delta D \; i \tag{S1}$$

where Δf is the frequency shift (a real number), f_F is the fundamental frequency, n_0 is the overtone number, ΔD is the dissipation shift and i is the imaginary number.

The frequency and dissipations shifts are explicitly given by[4]

$$\Delta f = -\frac{f_F}{\pi Z_q} \operatorname{Im}\left(\Delta Z_L^{*}\right)$$

$$\Delta D = \frac{2}{\pi Z_q n_0} \operatorname{Re}\left(\Delta Z_L^{*}\right) \tag{S2}$$

where ΔZ_L^{*} is the complex load impedance expressed relative to a reference state and Z_q is the acoustic impedance of the quartz sensor equal to 8.8×10^{6} kg m^{-2} s^{-1}.

ΔZ_L^* is defined as the ratio of the tangential stress (force per unit area) to the crystal surface velocity

$$\Delta Z_L^* = \sigma^* V_i^{*-1} = \frac{1}{S_i} \Delta F_i^* V_i^{*-1} \qquad \text{(S3)}$$

where σ^* is the complex tangential stress component, V_i^* is the complex sensor tangential velocity, S_i is the surface area of the sensor and ΔF_i^* is the excess tangential force acting on the sensor.

The force is opposite in direction to the net force acting on particles in the vicinity of the sensor. It consist for rigid particles of the hydrodynamic, the inertia and the specific surface contributions such as electrostatic and van der Waals forces. It should be mentioned that the specific forces have both the perpendicular and tangential components, the former fix the equilibrium particle distance from the surface, whereas the latter govern the rigidity of the particle contact with the surface. For larger particles the inertia and hydrodynamic forces may exceed the specific ones that can promote their rocking and sliding motion.

In order to calculate the hydrodynamic force one should first know the ambient flow in the vicinity of the oscillating sensor considered to be a flat plate. As shown in Ref.[5] the laminar flow relative to the plate ΔV is given by

$$\Delta V(z,t) = V_0\, e^{i\omega t}[e^{-(1+i)z/\delta} - 1] = V_i\, [e^{-(1+i)z/\delta} - 1] = V_i\, [e^{-z/\delta} \cos(z/\delta) - 1 - e^{-z/\delta} \sin(z/\delta) i] \qquad \text{(S4)}$$

where z is the distance perpendicular to the plate (see Figure S1), t is the time, $V_0 = \omega\, a_m$, $\omega = 2\pi f_F n_0$ is the angular velocity of the sensor, a_m is the amplitude of oscillations, $V_i = V_0\, e^{i\omega t}$ is the plate tangential velocity and

$$\delta = \left(\frac{2\nu}{\omega}\right)^{1/2} \qquad \text{(S5)}$$

is the hydrodynamic boundary layer thickness also referred to as the flow penetration depth[3-5], and ν is the kinematic viscosity of the liquid.

For distances from the plate much smaller than the penetration depth, where $z/\delta < 1$, the flow pattern simplifies to

$$\Delta V(z,t) = -V_i \ (1+i) z / \delta \tag{S6}$$

This is a simple shear flow with the rate given by

$$G = \frac{\partial V}{\partial z} = -V_i \ \frac{1+i}{\delta} \tag{S7}$$

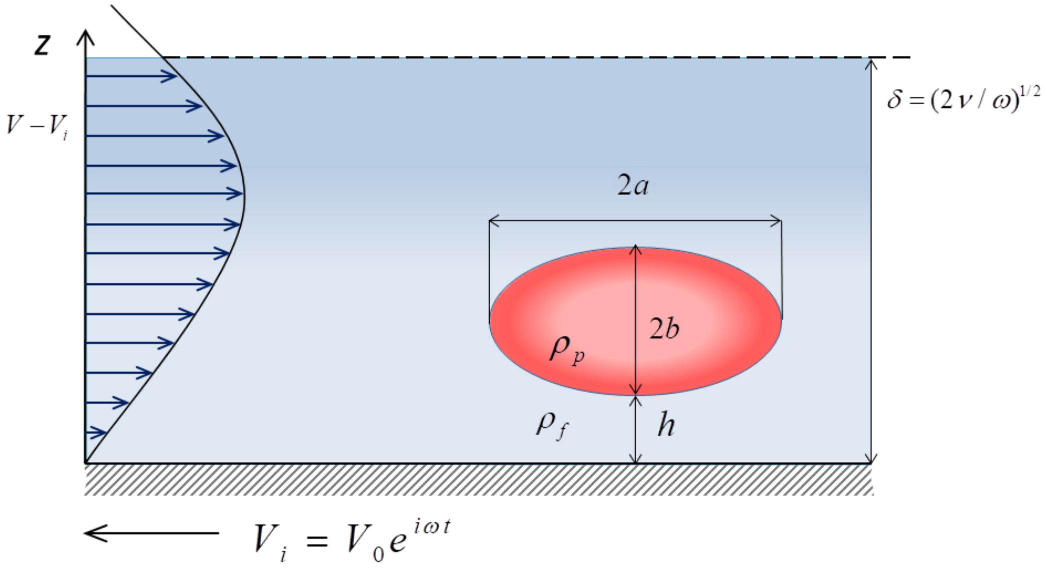

Figure S1. Schematic representation of the flow pattern near a spheroid particle in the vicinity of a oscillating solid boundary (QCM sensor)

It should be observed that the relaxation time of establishing the flow pattern given by Eq.(S4) can be calculated as

$$\tau = \frac{\delta^2}{\nu} \tag{S8}$$

Therefore, the flow at the distance $z < \delta$ is quasi-stationary, characterized by negligible explicit time dependence.

Knowing the flow pattern, the parallel component of the hydrodynamic force on a single spheroidal particle in the vicinity of the sensor (see Figure S1) can be calculated as[6]

$$F_h^* = 6\pi\rho_f \nu b(\Delta V - U) F_c(h/b, \lambda, \alpha) \tag{S9}$$

where ρ_f is the density of the liquid, b is the shorter semi-axis of the spheroid, U is the tangential component of the particle velocity due to hydrodynamic forces and torques and F_c is the correction function accounting for the presence of the wall depending on the surface to surface distance h and the spheroidal particle shape defined by the parameter $\lambda = a/b$. For prolate spheroids F_c also depends on the orientation of the spheroid longer axis against the flow direction, governed by the angle α.

In the general case, the hydrodynamic force can only be calculated by elaborated numerical techniques such as the Lattice Boltzmann method[7,8] or the immersed boundary method (IBM)[9,10]. However, useful analytical solutions can be derived for some limiting cases discussed below.

2.2. Force on rigidly fixed particles

For a stationary spherical particle where U = 0, with its center located at the distance $z = b + h$ much smaller than the penetration depth (see Figure S1) the hydrodynamic force calculated using Eqs.(S6,S9) is given by

$$F_h^* = -\omega\, m_p \frac{9}{4}\frac{\rho_f}{\rho_p}\frac{\delta}{b} F_8(h/b) V_i (1+i) \tag{S10}$$

where m_p is the particle mass, ρ_f, ρ_p are the fluid and particle densities, respectively, $F_8(h/b)$ is the universal hydrodynamic function pertinent to a spherical particle in a simple shear flow[11,12]. For a particle touching the sensor where h = 0, $F_8(0)$ is equal to 1.7005 [11] and for h/b =0.128, F_4 = 1.6682 and for h/b =0.5431, F_4 = 1.439 [12].

It is shown in Ref. [13,14] that for linear strings of touching spheres the $F_8(0)$ function was equal to 1.33, 1.14 and 1.01 for the number of spheres n_s equal to 2, 4 and 10, respectively.

On the other hand, in Ref. [15] the hydrodynamic force on the ensemble of N_p particles forming a rigid layer at the sensor was calculated applying the multipole expansion method. The numerical calculations were interpolated by the following analytical expression

$$F_i(N) = \frac{1 - e^{-C_i^o S_g N}}{C_i^o S_g N} \tag{S11}$$

where $N = N_p/S_i$ is the surface concentration of particles, $C_i^o = 6F_8(0) = 10.21$ is the dimensionless constant and S_g i the characteristic cross-section area of the particle, equal to πb^2 for spheres and πab for prolate spheroids.

For $N \to 0$, the $F_i(N)$ function approaches unity and for $S_g N = 0.5$, $F_i(N) = 0.196$.

It was also shown in Ref. [15] that the correction function is independent of the structure of the particle layer for the coverage up to 0.85.

In consequence the hydrodynamic force on a single particle in the layer is given by the formula

$$F_h^* = -\omega\, m_p \frac{9}{4}\frac{\rho_f}{\rho_p}\frac{\delta}{b} F_i(N) F_8(0) V_i (1+i) \tag{S12}$$

Interesting analytical solution was also derived in Ref.[4] in the opposite limit where the hydrodynamic boundary layer becomes much smaller than the particle radius, which is the case for larger particles and large oscillation frequency (overtone number). Under such conditions one can assume that the particle attached to the interface effectively oscillates in a stagnant fluid. In consequence, to calculate the hydrodynamic force, one can use the known solution discussed in Ref.[16]. In consequence, as shown in Refs.[4,5] the hydrodynamic force on the particle is given by

$$F_h^* = -\omega\, m_p [\frac{\rho_f}{\rho_p}\left(\bar{\delta} + \frac{9}{4}\bar{\delta}^2\right) + \frac{\rho_f}{\rho_p}\left(\frac{1}{2} + \frac{9}{4}\bar{\delta}\right) i]\ V_i \tag{S13}$$

where $\bar{\delta} = \delta / b$

One should remember that in the case of a fixed particle, the net force consist of the hydrodynamic and the inertia contributions, i.e.,

$$F^* = F_h^* - V_i \omega\, m_p i \tag{S14}$$

1.3 Force on neutrally buoyant particle

In Ref.[12] the translational and the rotational motion of a neutrally buoyant spherical particle immersed in a simple shear flow V_s = $G\,z$ near a solid wall was analyzed. The exact numerical solutions obtained in Ref.[12] yielded the particle tangential velocity as a function of h

$$U = GbF_3(h/b) \tag{S15}$$

where $F_3(h/b)$ is the universal correction function interpolated as [17]

$$F_3(h/b) = \frac{1}{0.754 - 0.256\ln(h/b)} \quad ; \text{valid for } h/b \leq 0.15$$

(S16)

$$F_3(h/b) = 1 - \frac{0.304}{(1+h/b)^3} \quad ; \text{valid for } h/b > 0.15$$

One can predict from Eqs.(S15,S16) that the normalized slip velocity of the particle relative to the ambient shear flow defined as $(\Delta V$ - $U)$ / ΔV is equal to 0.526, 0.43, and 0.255 for h/b = 0.005, 0.02 and 0.1. This indicates that even at distance between the particle surface and the solid wall (sensor) equal to 0.5% of b the hydrodynamic forces is not sufficient to fully immobilize the particle. It is interesting to mention that such an effect was numerically confirmed in Ref.[9].

Using Eqs.(S9,S15) the hydrodynamic force in the case of a neutrally buoyant particle can be expressed as

$$F_h^* = -\omega\, m_p \frac{9}{4}\frac{\rho_f}{\rho_p}\bar{\delta}\, \omega\, m_p[1 - F_3(h/b)]F_8(0)V_i(1+i) \tag{S17}$$

Because of the quasi-stationary motion assumption, Eq.(S17) remains exact if the particle semi-axis (radius) b remains is smaller than the penetration depth δ.

Considering the above results one can predict that the force exerted on the neutrally buoyant particle moving near a boundary in shear flow is considerably lower than that the force exerted on a stationary one. For example, at the distance h equal to 10% of the particle radius, the force is more than four time smaller.

In Ref.[18] analogous problem of a free spherical particle motion parallel to a solid wall in a quiescent fluid was considered. The exact numerical solutions were interpolated as follows

$$U = U_0 F_4(h/b) \tag{S18}$$

where $U_0 = V_i$ is the uniform translation velocity of the particle far from the wall and $F_4(h/b)$ is the universal correction function, which can be approximated as[17]

$$F_4(h/b) = \frac{1}{0.9588 - (8/15)\ln(h/b)} \quad ; \text{valid for } h/b \le 0.1$$

(S19)

$$F_4(h/b) = [\frac{h/b}{(2.64 + h/b)}]^{1/4} ; \text{valid for } h/b > 0.1$$

One can calculate from Eqs.(S18,S19) that in this case the normalized particle slip velocity relative to the bulk $(U_0 - U)/U_0$ is equal to 0.736, 0.672, and 0.543 for *h/b* = 0.005, 0.02 and 0.1. This indicates that the hydrodynamic coupling with the wall is much stronger compared to the particle motion in shear flow.

Using Eqs.(S18,S19) one can formulate the following expression for the hydrodynamic force on a freely oscillating particle in the case where $\bar{\delta} < 1$

$$F_h^* = -\omega\, m_p [1 - F_4(h/b)]\ [\frac{\rho_f}{\rho_p}\left(\bar{\delta} + \frac{9}{4}\bar{\delta}^2\right) + \frac{\rho_f}{\rho_p}\left(\frac{1}{2} + \frac{9}{4}\bar{\delta}\right) i]\ V_i \tag{S20}$$

The above derived expressions for the hydrodynamic force enable to explicitly calculate the frequency and dissipation shifts using the constitutive Eq.(S3) by noting that the excess tangential force acting on the sensor ΔF_i^* is opposite to the net force acting on the particle.

Thus, in the case of stationary (fixed) spheres, the frequency and dissipation shifts due to a layer of N_p particles of equal size calculated from Eqs.(S12,S14) are

$$\Delta f = -\frac{f_F \omega m_p}{\pi Z_q} N \operatorname{Im}\left(\Delta Z_L^*\right) = -\frac{n_0}{C_s} N\ m_p \left(1+\frac{9}{4}\frac{\rho_f}{\rho_p}\bar{\delta}\, F_i\left(N\right) F_8(0)\right)$$

(S21)

$$\Delta D = \frac{2\omega m_p}{n_0 \pi Z_q} N \operatorname{Re}\left(\Delta Z_L^*\right) = \frac{2}{f_F C_s} N\ m_p \left(\frac{9}{4}\frac{\rho_f}{\rho_p}\bar{\delta}\, F_i\left(N\right) F_8(0)\right)$$

where $N = N_p / S_i$ is the surface concentration of deposited particles (number of particles per unit area of the sensor) and

$$C_s = \frac{Z_q}{2 f_F^2} \tag{S22}$$

is the Sauerbrey constant equal to 0.177 (mg m^{-2}) for $f_F = 5\times 10^6$ Hz.

The quantity $N\, m_p$ in Eq.(S21) is the mass of the particle layer per unit area referred to in the QCM literature as the dry mass coverage and denoted hereafter by Γ.

It is to mention, however, that Eq.(S21) remains accurate for $\bar{\delta} > 1$. For the opposite case where $\bar{\delta} \le 1$ one obtain the following expression for the frequency and dissipation shifts due to a layer of N_p single particles

$$\Delta f = -\frac{n_0}{C_s} N\ m_p \left(1+\frac{\rho_f}{\rho_p}\left(\frac{1}{2}+\frac{9}{4}\bar{\delta}\right) F_{i2}\left(N\right)\right)$$

(S23)

$$\Delta D = \frac{2}{f_F C_s} N\ m_p \left(\frac{\rho_f}{\rho_p}\left(\bar{\delta}+\frac{9}{4}\bar{\delta}^2\right) F_{i2}\left(N\right)\right)$$

where $F_{i2}\left(N\right)$ is the correction function, which is expected to be close to unity for the entire range of particle coverage.

Using Eq.(S17) one can derive analogous dependencies for the layer of neutrally buoyant particles and $(b+h)/\delta > 1$

$$\Delta f = -\frac{n_0}{C_s} N\ m_p \left(\frac{9}{4}\frac{\rho_f}{\rho_p}\bar{\delta}\, F_{ib}\left(N\right)[1-F_3(h/b)]F_8(0) \right)$$

$$\Delta D = \frac{2}{f_F C_s} N\ m_p \left(\frac{9}{4}\frac{\rho_f}{\rho_p}\bar{\delta}\, F_{ib}\left(N\right)[1-F_3(h/b)]F_8(0) \right) \tag{S24}$$

where $F_{ib}\left(N\right)$ is the correction function.

For the opposite case where $(b+h)/\delta \le 1$ one obtains from Eq.(S20) the following expression for the frequency and dissipation shifts due to a layer of N_p single particles

$$\Delta f = -\frac{n_0}{C_s} N\ m_p \left(\frac{\rho_f}{\rho_p}\left(\frac{1}{2}+\frac{9}{4}\bar{\delta}\right) F_{ib2}\left(N\right)[1-F_4(h/b)] \right)$$

$$\Delta D = \frac{2}{f_F C_s} N\ m_p \left(\frac{\rho_f}{\rho_p}\left(\bar{\delta}+\frac{9}{4}\bar{\delta}^2\right) F_{ib2}\left(N\right)[1-F_4(h/b)] \right) \tag{S25}$$

where $F_{ib2}\left(N\right)$ is the correction function, which is expected to be close to unity for the entire range of particle coverage.

2 Modeling Adsorption Kinetics of Particles in the QCM cell.

Particle deposition kinetics under convective-diffusion transport conditions at solid substrates (for example QCM sensor) can be theoretically described using a hybrid approach exploiting the convective-diffusion equation [17]

$$\frac{\partial n}{\partial t} = D\ \nabla^2 n - \frac{D}{kT}\nabla\cdot\left(\mathbf{F}\, n\right) - \mathbf{V}\cdot\nabla n \tag{S26}$$

where n is the number concentration of particles, t is the time, D is the translation diffusion coefficient, k is the Boltzmann constant, T is the absolute temperature, **F** is the external force vector and **V** is the unperturbed (macroscopic) fluid velocity vector.

Eq.(S26) is coupled with the surface layer transport equation where the fluid convection effects are neglected [17,19]

$$j_a = \frac{dN}{dt} = k_a\, n(\delta_a)\; B(N) - \frac{k_d}{S_g} N \tag{S27}$$

where j_a is the net adsorption/desorption flux, k_a, k_d are the adsorption and desorption constants, $n(\delta_a)$ is the number concentration of particles at the adsorption boundary layer of the thickness δ_a and $B(N)$ is the generalized blocking function.

Under convective transport, where the particle concentration $n(\delta_a)$ remains in a local equilibrium with the surface coverage, the constitutive expression for the adsorption flux, Eq.(S27) can be expressed as [17]

$$j_a = \frac{K\,B(N) - K_d N}{(K-1)B(N)+1} k_c n_b \tag{S28}$$

where $K = k_a / k_c$ is the dimensionless coupling constants, $K_d = k_d/(S_g k_c n_b)$ is the dimensionless desorption constant, k_c is the bulk transfer rate constant, known in analytical form for many types of flows and n_b is the bulk number concentration of particles.

Eqs.(S27,S28) can be expressed in the form of the definite integral

$$\int_{\Gamma_0}^{\Gamma} \frac{(k_\mathrm{a} - k_c) B(\Gamma') + k_c}{k_a c_b B(\Gamma') - k_d \Gamma'} d\Gamma = k_\mathrm{c} t \tag{S29}$$

where $\Gamma = N\, m_\mathrm{p}$ is the mass coverage of particles, Γ_0 is the initial coverage, and $c_b = m_\mathrm{p}\, n_b$ is the mass concentration of particles in the bulk.

Eq.(S29) represents a general solution for particle deposition kinetics under convection driven transport. However, it can only be evaluated by numerical integration methods if the blocking function is known in an analytical form [17,19]. For bulk transport controlled regime characterized by the condition $k_a >> k_c$ and a lower coverage range, Eq. (S29) simplifies to the linear form

$$\Gamma = k_c c_b t \tag{S30}$$

The adsorption and the desorption constants in Eq.(S30) can be calculated from the DLVO energy profiles, if the particle and sensor zeta potential are known, as well as the Hamaker constant[17]. For a barrier-less regime pertinent opposite zeta potentials of particles

and the sensor, there appears a deep energy minimum, therefore, $k_d = 0$ and the kinetic adsorption constant can be calculated from the dependence[17,19]

$$k_a = \frac{D}{\delta_a[1+0.5\ \ln(\delta_a/\delta_m)]} \tag{S31}$$

where δ_m is the primary minimum distance.

In order to explicitly calculate particle deposition kinetics from Eq.(S31) one should also know the blocking function, which can be conveniently acquired from the random sequential adsorption (RSA) modeling[17,20,21]. For not too large coverage range, one can approximate the blocking function by the second order series expansion

$$B(\Theta) = 1 - C_1\Theta + C_2\Theta^2 + 0\left(\Theta^3\right) \tag{S32}$$

For spheres $C_1 = 4$ and $C_2 = 6\sqrt{3}/\pi = 3.31$.

The analytical results calculated from Eq.(S32) agree with exact data derived from RSA simulations for the lower coverage range, if $\Theta < 0.3$.

On the other hand, for coverages approaching the jamming coverage Θ_∞, the blocking function for spheres can be approximated by the expression

$$B(\Theta) = 2.31\left(1 - \frac{\Theta}{\Theta_\infty}\right)^3 \tag{S33}$$

In the case of spheres, one can also formulate an analytical expression fitting well the exact numerical data for the entire range of coverage[21]

$$B(\Theta) = \left[1 + 0.812\bar{\Theta} + 0.4258\left(\bar{\Theta}\right)^2 + 0.0716\left(\bar{\Theta}\right)^3\right]\left(1-\bar{\Theta}\right)^3 \tag{S34}$$

where $\bar{\Theta} = \frac{\Theta}{\Theta_\infty}$.

For the side-on adsorption of prolate spheroidal particles, the blocking function at the jamming limit assumes an analogous form

$$B(\Theta) = C_\infty\left(1 - \frac{\Theta}{\Theta_\infty}\right)^4 \tag{S35}$$

where the dimensionless constant C_∞ varies between 2.8 - 3.2 for spheroids[21]

It was shown in Ref.[17] that the above results obtained pertinent to hard particles can also be extended to the case of particles interacting via the short- range Yukawa potential. For electrostatic double-layer interactions the characteristic range of this potential is given by

$$h^* = \frac{Le}{d_p}\left[\ln\frac{\phi_o}{\phi_{ch}} - \ln\left(1+\frac{Le}{d_p}\ln\frac{\phi_o}{\phi_{ch}}\right)\right] \qquad \text{(S36)}$$

where Le is the electric double layer thickness, d_p is the particle characteristic dimension, ϕ_o is electrostatic energy at contact and ϕ_{ch} is the characteristic interaction energy.

Consequently, one can calculate the jamming coverage for interacting particles referred to as the maximum coverage) from the relationship

$$\Theta_{mx} = \Theta_\infty \frac{1}{(1+h^*)^2} \qquad \text{(S37)}$$

Knowing Θ_{mx} one can use Eq.(S35) to calculate the blocking function substituting $\overline{\Theta} = \frac{\Theta}{\Theta_{mx}}$

References

1. Bratek-Skicki, A.; Sadowska M.; Maciejewska-Prończuk J.; Adamczyk, Z. *Nanomaterials,* **2021**, 11, 145, 1-38.
2. Sauerbrey, G. *Z. Phys.* 1**959**, 155, 206–222.
3. Johannsmann, D. *Phys. Chem. Chem. Phys.* **2008**, 10, 4516-4534.
4. Tarnapolsky, A.; Freger, V. *Anal. Chem.* **2018**, 90, 13960-13968.
5. Adamczyk, Z.; Sadowska, M. *Anal. Chem*. **2020**, 92, 3896-3903.
6. Happel, J.; Brenner, H, *Martinius Nijhoff Publishers,* Kluwer, **1983**.
7. Gillissen, J.J.J.; Jackman, J.A.; Tabaei, S.R.; Yoon, B.K.; Cho, N.-J. *Anal. Chem.* **2017**, 89, 11711-11718.
8. Goplakrishna, S.; Langhoff, A.; Brenner, G.; Johansmann, D. *Anal. Chem*. **2021**, 93, 10229- 10235.
9. Melendez-Schofiel, M.; Delgado-Buscalioni, R.D. *Soft Matter*. **2021**, 17, 8160-8174.
10. Melendez M.; Vasquez-Queseda A.; Delgado-Buscalioni, R.D. *Langmuir*. **2020**, 36, 9225-9234.
11. O'Neill, M.E. *Chem. Eng. Sci.* **1968**, 23, 1293-1298.
12. Goldman A.J.; Cox R.G.; Brenner H. *Chem. Eng. Sci.* **1967**, 22, 653-660.
13. Ekiel-Jeżewska, M.L.; Sadlej, K.; Wajnryb, E. *J. Chem.Phys.* **2008**, 129, 041104-1-4.
14. Adamczyk, Z.; Sadlej, K.; Wajnryb, E.; Nattich, M.; Ekiel-Jeżewska, M.L.; Bławzdziewicz, J. *Adv. Colloid Interface Sci.* **2010**, 153, 1-29.
15. Ekiel-Jeżewska, M.L; Adamczyk Z.; Bławzdziewicz J. *J. Phys. Chem. C*. **2019**, 123, 3517-3531.

16. Pozrikidis C., *Introduction to Theoretical and Computational Fluid Dynamics*, Oxford University Press, **1997**.
17. Adamczyk, Z. *Particles at Interfaces*, Elsevier, Academic Press, **2017**.
18. Goldman A.J.; Cox R.G.; Brenner H. *Chem. Eng. Sci.* **1967**, 22, 637-651.
19. Adamczyk, Z. *J. Colloid Interface Sci*. **2000**, 229, 477-489.
20. Talbot, J.; Tarjus G.; van Tassel, P.R.; Viot, P. *Colloids Surf., A*. **2000**, 165, 287-324.
21. Ricci, S.M.; Talbot, J.; Tarjus, G.; Viot, P. *J. Chem. Phys*. **1992**, 97, 5219-5228.
